# A predictive model for household displacement duration after disasters

**DOI:** 10.1111/risa.17710

**Published:** 2025-02-25

**Authors:** Nicole Paul, Carmine Galasso, Jack Baker, Vitor Silva

**Affiliations:** ^1^ Institute for Risk and Disaster Reduction University College London London UK; ^2^ Department of Civil, Environmental and Geomatic Engineering University College London London UK; ^3^ Department of Structural Engineering and Geomechanics Stanford University Stanford California USA; ^4^ Global Earthquake Model Foundation Pavia Italy

**Keywords:** disaster displacement, disaster risk, displacement duration, household displacement, machine learning, population return

## Abstract

According to recent Household Pulse Survey data, roughly 1.1% of households were displaced due to disasters in the United States in recent years. Although most households returned relatively quickly, 20% were displaced for longer than 1 month, and 14% had not returned by the time of the survey. Protracted displacement creates enormous hardships for affected households and communities, yet few disaster risk analyses account for the time component of displacement. Here, we propose predictive models for household displacement duration and return for practical integration within disaster risk analyses, ranging in complexity and predictive power. Two classification tree models are proposed to predict return outcomes with a minimum number of predictors: one that considers only physical factors (TreeP) and another that also considers socioeconomic factors (TreeP&S). A random forest model is also proposed (ForestP&S), improving the model's predictive power and highlighting the drivers of displacement duration and return outcomes. The results of the ForestP&S model highlight the importance of both physical factors (e.g., property damage and unsanitary conditions) and socioeconomic factors (e.g., tenure status and income per household member) on displacement outcomes. These models can be integrated within disaster risk analyses, as illustrated through a hurricane scenario analysis for Atlantic City, NJ. By integrating displacement duration models within risk analyses, we can capture the human impact of disasters more holistically and evaluate mitigation strategies aimed at reducing displacement risk.

## 1 INTRODUCTION

According to the United States Household Pulse Survey (HPS), approximately 1.1% of American households were displaced due to disasters between December 2021 and July 2024. Most of these households returned quickly: 43% within 1 week and another 23% within 1 month. However, 20% of households took over 1 month to return, and 14% had not returned by the time of answering the survey. Protracted displacement is associated with profound negative consequences for households, including job loss, disruption of education and its long‐term effects on children, and increased post‐traumatic stress or other psychosocial effects (e.g., Blaze & Shwalb, 2009; Bolin, 1982; Hori & Schafer, 2010; Picou & Marshall, 2007). When affecting multiple households simultaneously, consequences can ripple out into the larger community (e.g., outmigration and urban blight, lost economic production, and broader economic impact).

Despite the importance of displacement duration, most of the data collected after disaster events are biased toward the emergency phase and evacuations, creating a false perception that displacement is relatively short‐lived (IDMC, 2019). Similarly, most disaster risk models focus on the drivers of initial displacement, when households may evacuate due to perceived risks (Dash & Gladwin, 2007) or dislocate when housing is rendered uninhabitable by damage, utility disruption, and weather conditions (Chang et al., 2008). In contrast, disaster risk models often neglect the drivers of long‐term displacement (e.g., resource availability and the pace of reconstruction) and permanent relocation (Costa, Haukaas, et al., 2022). The process of temporary displacement versus permanent relocation is distinct and relies upon household decisions to return, which is a more voluntary process, albeit constrained by moving costs, housing availability, and the proximity of livelihoods. These decisions to return or relocate have been shown to vary by a range of factors, such as homeownership status, insurance availability, place attachment, and income level. However, with a few exceptions (e.g., Burton et al., 2019; Costa, Wang et al., 2022), these factors are rarely incorporated in disaster risk models. Neglecting displacement duration in reported data and models creates ambiguity in disaster risk reduction policy and practice, as it mixes the benefits of potentially life‐saving and minimally disruptive short‐term evacuations with the negative impacts of prolonged displacement on affected households’ well‐being, livelihoods, and access to essential services (Guadagno & Yonetani, 2023). This study aims to fill this gap in disaster risk models by proposing evidence‐based and practical predictive models for displacement outcomes, which clearly distinguish between short‐term displacement, long‐term displacement, and potentially permanent relocation.

The recent introduction of questions about disaster displacement and return in the HPS offers an opportunity to fit data‐driven predictive models for household displacement durations and return after disasters. Explainable artificial intelligence (XAI) methods can then quantify the contribution of the considered factors (e.g., property damage, tenure status, and income level) on the model's predictions of different displacement durations, allowing an understanding of the different drivers of short‐term displacement, long‐term displacement, and potential permanent relocation. The range of uncertainty and variability across return outcomes can also be assessed by evaluating the predictive model's performance on a testing set that was not used to train the model.

This study fits existing machine learning algorithms to HPS data in order to propose novel predictive models for household displacement duration and return after disasters: two classification tree models and a random forest model. The classification tree models use fewer predictors and are thus relatively simple to integrate within disaster risk analyses. The random forest model is more complex but improves model performance and allows the quantification of each factor's contribution to each displacement duration prediction. Finally, a sensitivity study is performed for a Category 3 hurricane scenario in Atlantic City, NJ, to demonstrate the applicability of the proposed models in practice and assess the impact of considering only physical factors (i.e., the standard practice in disaster risk analyses; Sutley et al., 2019) versus additionally considering socioeconomic factors.

## 2 THE UNITED STATES HOUSEHOLD PULSE SURVEY

This section introduces the United States HPS dataset, provides key descriptive statistics, and describes the potential predictors of disaster displacement durations within the HPS in combination with a literature review.

### 2.1 Overview of the dataset

During the onset of the coronavirus pandemic in 2020, the United States Census Bureau initiated a 20‐min online household survey called the Household Pulse Survey (HPS) to study the pandemic and other emergent issues (e.g., infant formula access, employment, and mental health). In December 2022, questions regarding disaster displacement were introduced to the HPS, such as:

- “In the past year, were you displaced from your home because of a natural disaster?”
- “What type of natural disaster?”
- “How long were you displaced from your home?”
- “Altogether, how much damage to your property or possessions did you experience as a result of natural disasters in the last year?”
- “In the first month after the natural disaster, to what extent did you experience any of the following: a shortage of food, a shortage of drinkable water, a loss of electricity, unsanitary conditions (e.g., inadequate toilets), feeling isolated, fear of crime, or offers that seemed like a scam?”

At the time of writing, 19 cycles of household‐level responses that include the disaster displacement questions have been released as public use files (PUF), spanning 19 months of data collection and representing 1,326,503 individual households. Since the survey asks about disasters experienced within the last year, the disaster period considered theoretically spans from December 9, 2021, to July 22, 2024.

### 2.2 Descriptive statistics

**FIGURE 1:**
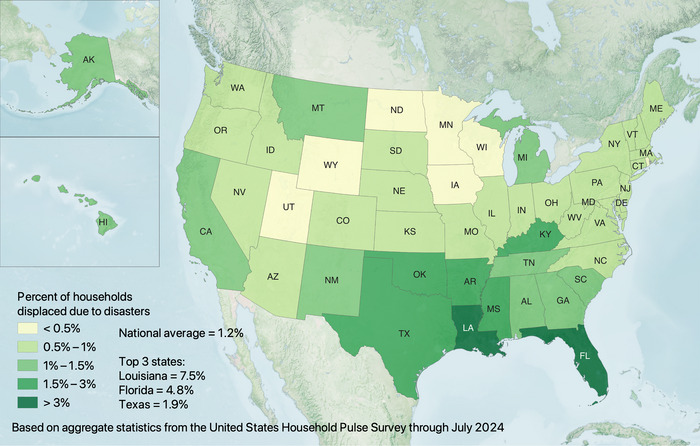
Percentage of households displaced by state according to the United States Household Pulse Survey (based on all available survey data where displacement is included through July 2024).

Based on data available through July 2024, 1.12% of American households were displaced due to disasters. However, the rate varies state by state, with households in Louisiana and Florida being about 6.8 and 4.4 times more likely to be displaced than the national average, respectively (see Figure 1).

Hurricanes are the most common disaster type cited by displaced households, followed by an “other” category that likely encompasses hazards such as winter storms, other types of severe weather, and earthquakes. Table 1 shows the breakdown of disaster types.

**TABLE 1:** Representation of hazard types per the United States Household Pulse Survey (based on all available through July 2024).

Disaster type	Sample size	Proportion (%)
Hurricane	3653	27.8
Flood	2463	18.8
Fire	2078	15.8
Tornado	1445	11.0
Other	3490	26.6

**FIGURE 2:**
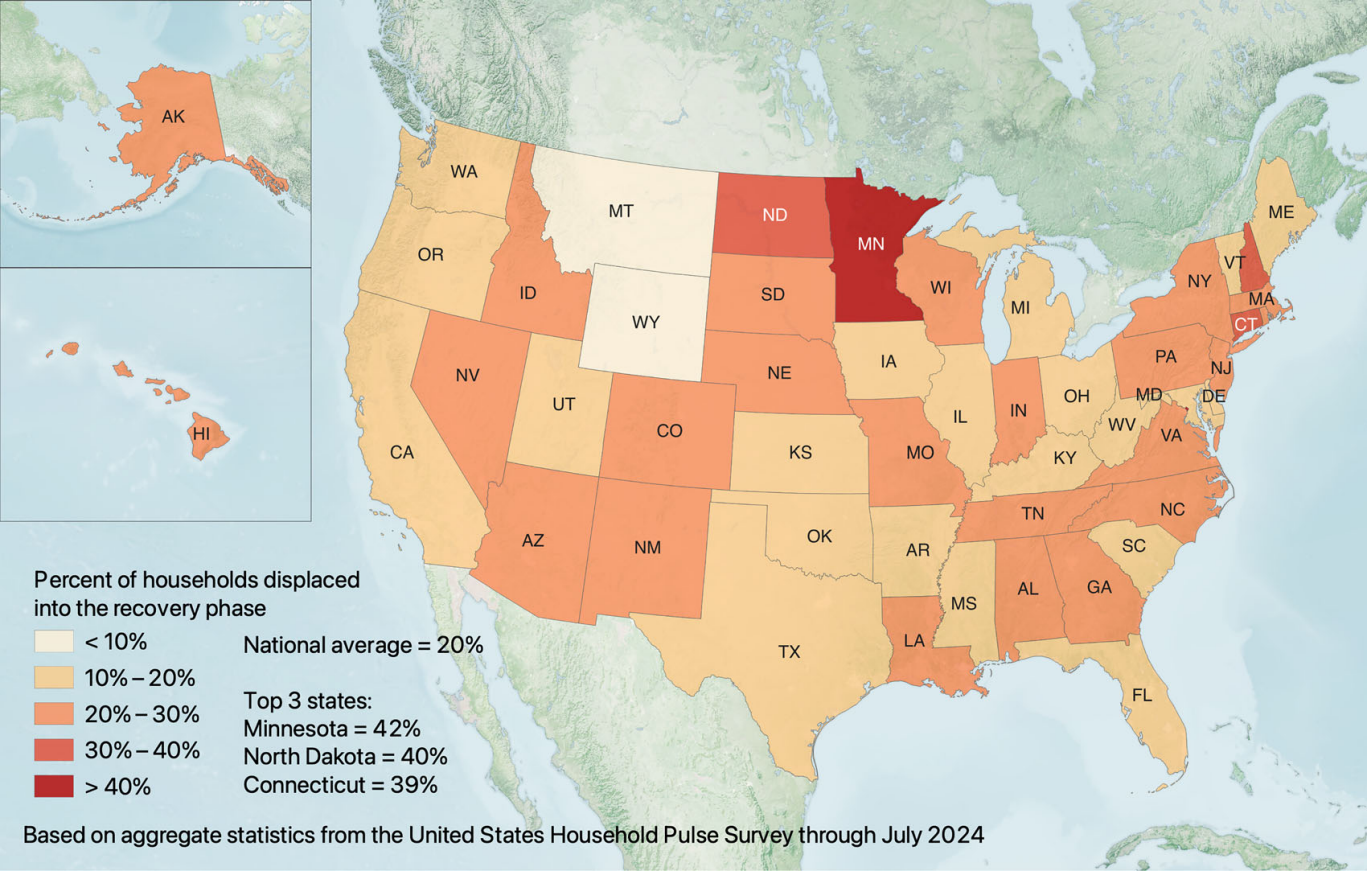
Percentage of displaced households per state that returned in the recovery phase (i.e., took longer than 1 month to return) according to the United States Household Pulse Survey (based on all data where displacement is included through July 2024).

**FIGURE 3:**
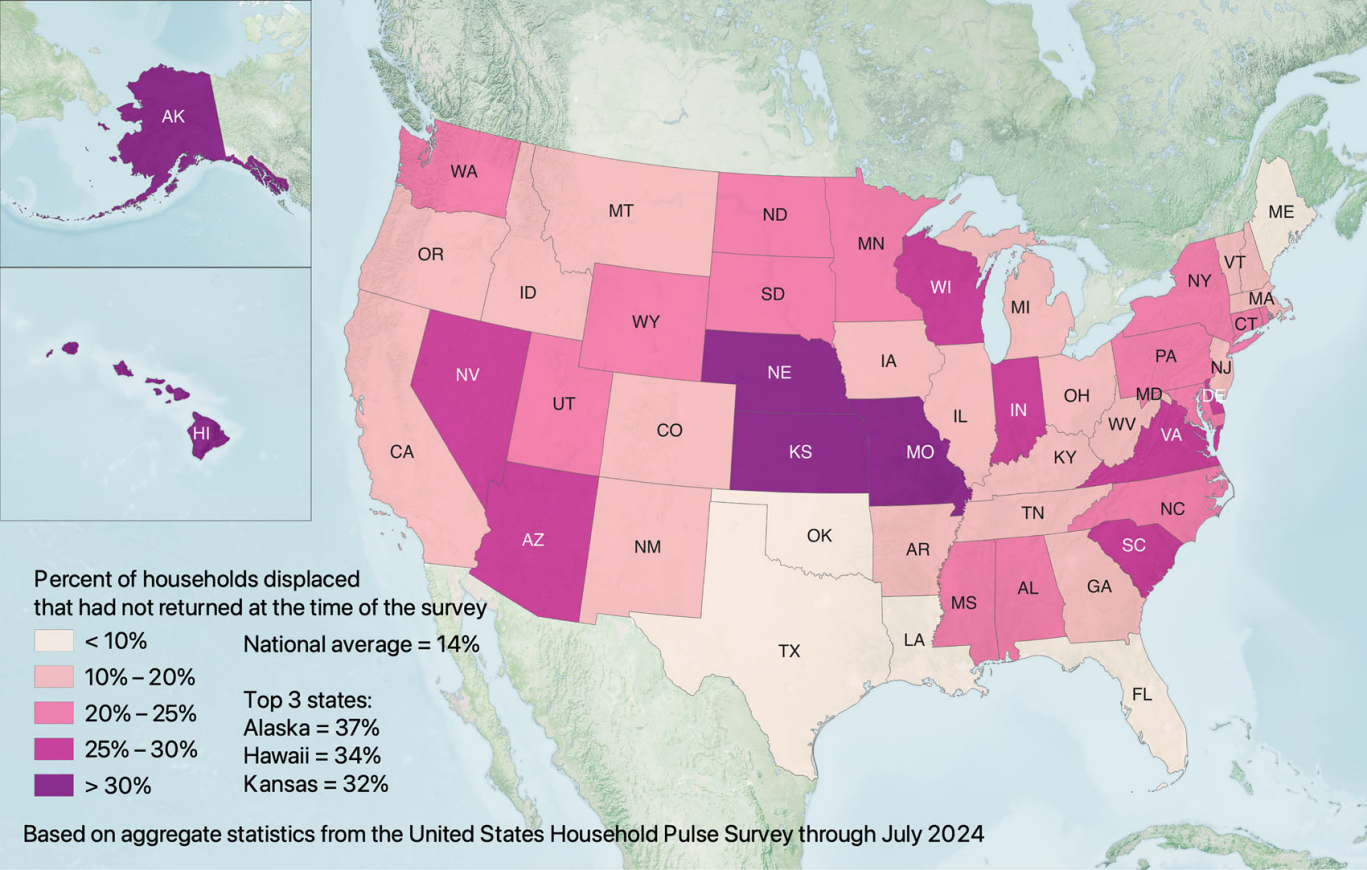
Percentage of displaced households per state that had not returned according to the United States Household Pulse Survey (based on all data where displacement is included through July 2024).

Although Louisiana and Florida experienced the highest rates of disaster displacement, they did not have the most prolonged displacement durations. Instead, displaced households in states such as Minnesota, North Dakota, and Connecticut were considerably more likely to take longer than 1 month to return (see Figure 2). Further, displaced households in states such as Alaska, Hawaii, and Kansas were considerably less likely to return than in Louisiana and Florida (see Figure 3).

### 2.3 Potential predictors of displacement duration in the HPS

The HPS also includes data on several potentially relevant factors that explain or predict different displacement and return outcomes (e.g., tenure, income level, and dwelling type). According to a literature review of household displacement and return (Paul et al., 2024), the determinants of return decisions can be grouped into the following categories: physical damage to the built environment, household demographics, psychological and social phenomena, and pre‐ and post‐disaster policies. The variables included in the HPS PUFs primarily cover the categories of physical damage to the built environment (see Table 2) and household demographics (see Table 3).

**TABLE 2:** Overview of the explanatory variables that quantify physical damage to the built environment.

Variable(s)	Justification
Property damage	Housing damage has consistently been identified as a primary driver of displacement following disasters (e.g., Burton et al., 2019; Comerio, 1997; Cong et al., 2018; Mayer et al., 2020; Scheele et al., 2020; Smith & McCarty, 1996). A common assumption is that damage incurred during a disaster can render housing “uninhabitable,” displacing residents. Further, housing damaged to a higher degree takes longer to recover due to the complexity and cost of repairing severe damage or demolition and replacement.
Water shortage; electricity loss	Utility disruption can also render housing “uninhabitable” (e.g., Chang et al., 2008; Costa, Haukaas, et al., 2022). For example, loss of water can inhibit daily domestic functions (e.g., cooking and potable water access), and electricity loss can generate a range of complications for daily life (e.g., inability to keep medication and food properly refrigerated, and thermal comfort issues due to a lack of air conditioning or heating).
Unsanitary conditions	Post‐disaster unsanitary conditions may prompt households to evacuate or delay their return. This includes the consequences of disrupted sewage systems (e.g., inadequate toilets), contaminants in floodwaters and mold, the release of pollutants from nearby damaged refineries/plants, and disease spread due to crowded conditions in shelters (e.g., Bhandari et al., 2022; Chow et al., 2019; Du et al., 2010; Matsuhashi et al., 2014; Noji, 1997). Landlord concern over liability for unsanitary conditions has also been indicated as a potential reason for the increased evictions after Hurricane Michael in Florida (Brennan et al., 2022).
Food shortage	Food security (i.e., the reliability, quantity, and affordability of nutritious food) can be compromised after disasters due to supply chain disruptions and agricultural losses, financial hardships for lower income households during post‐disaster competition for resources, and contamination and spoilage due to utility disruption and/or unsanitary conditions (e.g., Cutter, 2017; MacNabb & Fletcher, 2019). Food‐insecure households may struggle to afford moving costs, temporary accommodation, and potential rental price increases.
Hazard type	Different natural hazards produce different damage extents within a community (e.g., tornadoes are more localized than hurricanes). Additionally, the type of damage suffered due to hazard effects (e.g., hurricane‐induced wind and earthquake‐induced ground shaking) can vary. Since most existing literature on disaster displacement and return comprises case studies of individual events (e.g., Girard & Peacock, 1997; Groen & Polivka, 2010; Henry, 2013), the extent to which hazard type may influence the determinants of household return is relatively unstudied. If hazard type appears as a strong predictor in model fitting, it could be desirable to fit individual models for each hazard type separately.
Dwelling type	A consistent observation after disaster events is that single‐family homes recover faster than multi‐family homes, even after controlling for the damage level (e.g., Hamideh et al., 2021; Lu et al., 2007). The dwelling type can also act as a proxy for the repair or reconstruction time, a determinant of return identified in the literature review (Paul et al., 2024). Reasons for the prolonged reconstruction time of multi‐family residences include complex ownership structures that require time to build consensus on recovery decisions (e.g., Johnson & Olshansky, 2016; Wu & Lindell, 2004) and a bias of post‐disaster policies toward single‐family owner‐occupied homes in the United States (Comerio, 1997).

**TABLE 3:** Overview of the explanatory variables considered that quantify household demographics.

Variable(s)	Justification
Tenure status	Homeownership status is a consistent determinant of household return in disaster literature (e.g., Cong et al., 2018; Elliott & Pais, 2006; Kim & Oh, 2014; Y.‐J. Lee et al., 2017; Mayer et al., 2020). Homeowners may have higher levels of place attachment (Binder et al., 2015; Henry, 2013) or mortgage obligations (Elliott & Pais, 2006) that encourage return. Additionally, disaster assistance in the United States favors owner‐occupied single‐family homes (Comerio, 1997). Renters may also confront more obstacles: they have limited control over recovery decisions on their homes and have to compete over a limited number of affordable rental units if unable to enter their buildings (e.g., Comerio, 1998; Mukherji, 2014; Pardee, 2012).
Income per household member; educational attainment	There is consistent evidence in disaster literature that people with lower socioeconomic status (e.g., low‐income households and those with low educational attainment) more frequently inhabit hazard‐prone areas, reside in lower quality dwellings, and have lower access to risk reduction measures such as structural strengthening or insurance (e.g., Hallegatte et al., 2020; Tierney et al., 2001; Fothergill & Peek, 2004; Bolin & Stanford, 1991). As a result, households with low socioeconomic status may experience more significant damage in a disaster, increasing the likelihood of displacement and delayed return. Moreover, those with lower socioeconomic status are more likely to face obstacles in the recovery phase, such as challenges accessing assistance (e.g., Comerio, 2014; Dash et al., 1997; Peacock et al., 2018) and fewer resources to lessen the effects of displacement (e.g., to afford temporary lodging or price increases).
Age	Older individuals have been observed to have a higher likelihood of return after some disaster events (Cong et al., 2018; Groen & Polivka, 2010; Hu et al., 2019). One reason for this could be the desire to recoup their social supports (e.g., family, community resources, friends, and physicians; Sanders et al., 2004). However, older individuals are also more likely to live in low‐quality housing and experience higher relative loss due to limited financial means (e.g., Bolin & Klenow, 1988; Ngo, 2001), which could impede their ability to return quickly.
Race; Hispanic origin	Past studies on the role of race and ethnicity in household return in the United States have been mixed. For example, housing in neighborhoods with a higher percentage of minority residents (i.e., Blacks and Hispanics) recovered more slowly after the 1992 Hurricane Andrew in South Miami‐Dade County, Florida (Zhang & Peacock, 2009). In another study, the delayed return of Black residents to New Orleans after the 2005 Hurricane Katrina was found to be primarily due to differential housing damage and not necessarily race (Fussell et al., 2010; Groen & Polivka, 2010). At the same time, discriminatory practices can limit options for those seeking alternative accommodations (Girard & Peacock, 1997). Furthermore, distrust in the government, poor language skills, or lack of political and social capital can limit access to disaster assistance (e.g., Aldrich, 2010; Bolin & Stanford, 1998; Mukherji, 2010; Phillips, 1993; Prater & Lindell, 2000).
Household size; marital status	Family and relationships were identified as one of the top determinants of household return across a review of quantitative studies (Paul et al., 2024). For example, Nejat, Cong, and Liang (2016) found that those who lived with family members before the 2012 Hurricane Sandy in Staten Island, New York, were less likely to relocate than those who lived alone. There is evidence that partnered individuals have complex “linked lives,” whereby a larger number of motivators are required to prompt relocation (Coulter & Scott, 2015). Accordingly, those with larger household sizes were observed as slower to evacuate (Thompson et al., 2017). Larger households also relocated less after the 2013 Moore tornado in Moore City, Oklahoma, and 2012 Hurricane Sandy in Staten Island, New York (Hu et al., 2021; Hu & Nejat, 2021; Nejat et al., 2020).
School enrollment	Schools may encourage households to return in the interest of educational continuity. In contrast, closed schools can inhibit the desire to return (Peek et al., 2011). Additionally, school enrollment data have been used as a proxy for measuring longitudinal household displacement (e.g., Sharygin, 2021; Hinojosa and Meléndez, 2018; Newell, Beaven, and Johnston, 2012).
Employment status	Damage to businesses (e.g., Miles & Chang, 2011; Xiao & Van Zandt, 2012) and workplaces (Comerio, 1997) may discourage return, whereas job obligations may prevent households from leaving or prompt quicker return. Further, the loss of jobs or destruction of livelihoods can limit the ability of displaced households to return and recover (Elliott & Pais, 2006; Pu et al., 2021).
Limited mobility	Physically disabled individuals were less likely to evacuate after Hurricanes Bonnie, Dennis, and Floyd in North Carolina, primarily due to a perceived lack of access to services and assistance (Van Willigen et al., 2002). Additionally, households with a physically disabled member experienced more severe housing damage after those hurricanes (Van Willigen et al., 2002), which could cause dislocation or delayed return. Moreover, households with a disabled family member tend to have lower incomes (Jajtner et al., 2020), which can increase the number of obstacles faced (e.g., inability to afford transport or moving costs and challenges qualifying for post‐disaster loans).

Although HPS data do not directly cover psychological and social phenomena or pre‐ and post‐disaster policies, area attributes (e.g., vacancy rates and long‐term migration trends) can be included as proxies (see Table 4). In other cases, no appropriate proxies were identified. These are discussed further in Table 5.

**TABLE 4:** Overview of the explanatory variables that quantify psychological and social phenomena or pre‐ and post‐disaster policies.

Variable(s)	Justification
Population change (2010–2020)	Disasters often accelerate ongoing social, economic, and political trends (e.g., Haas et al., 1977; Kates et al., 2006). Accordingly, pre‐disaster community conditions or post‐disaster functioning can prompt displaced households to act upon previous desires or plans, thereby quickening existing migration trends (e.g., Belcher & Bates, 1983; Nawrotzki et al., 2014). As such, the baseline long‐term (10 years) migration trend is included as a proxy for the pre‐disaster community conditions.
Disaster declarations (2021–2023)	The total number of major disaster declarations per county within each state is included as a proxy for the compounding impacts of coincident and cascading hazards. Multiple consecutive disaster events complicate recovery trajectories: damage may accumulate, households affected by the first event may face additional obstacles and delays to return in the subsequent events, and assistance providers (e.g., insurance and the government) may become overwhelmed. For example, Louisiana was hit by Hurricanes Laura, Delta, and Zeta in 2020, prompting 1497 complaints to the Louisiana Department of Insurance by May 2021, where the most common grievance was delayed payments (Donelon, 2021). An additional 478,417 complaints were filed in 2021 after Hurricane Ida hit Louisiana (Louisiana Department of Insurance, 2022).
Homeowner vacancy rate; rental vacancy rate	Housing vacancy rates reflect the preexisting community conditions and policy choices (e.g., incentives for housing development, land annexation, housing market crashes, and economic downturns; Newman et al., 2019). The availability of affordable vacant units can also allow displaced households to remain nearby and/or to resettle into available units (Comerio, 1997; Mukherji, 2017). However, high vacancy rates could also signify other issues within the community (e.g., maintenance problems and a lack of local amenities).
Homeownership rate	Counties with higher homeownership rates were observed to have higher rates of evacuee return in low‐damage areas (but not high‐damage areas) after the 2005 Hurricane Katrina (Groen & Polivka, 2010). Homeowners are generally associated with higher return rates and quicker returns than renters (see “Tenure status” in Table 3). If areas with higher homeownership rates have higher rates of return, those areas are also less likely to be subject to the “snowball effect,” where more temporarily displaced households decide to relocate as other households in their neighborhood relocate (e.g., Costa, Haukaas, et al., 2022; Nejat & Damnjanovic, 2012).
Unemployment rate	The unemployment rate is another indicator of pre‐disaster community functioning (i.e., labor market conditions). Economic opportunity has long been considered a primary driver of residential mobility in non‐disaster conditions (e.g., Spring, Tolnay, and Crowder, 2016), but there has been limited study on its role in disaster migration.

**TABLE 5:** Overview of the topics that were not explicitly considered due to a lack of available data.

Topic	Elaboration
Overall community damage	Aside from housing damage, community‐wide damage to businesses, schools, services, and cultural assets could discourage return (e.g., Xiao and Van Zandt, 2012; Comerio, 1997; Airriess et al., 2008). Moreover, widespread damaged housing in the community could lead to a “snowball effect” where the relocations of neighboring households prompt other households to follow suit (e.g., Costa, Haukaas, et al., 2022; Nejat & Damnjanovic, 2012). Some aspects of community damage are explicitly considered (i.e., “Water shortage,” “Electricity loss,” and “Unsanitary conditions” in Table 2). However, other aspects are not (e.g., proportion of housing damaged, disruption of jobs or education). To some degree, the “Hazard type” relates to the spatial extent (e.g., tornadoes are more localized than hurricanes).
Reconstruction progress	Affected households may face prolonged displacement if housing remains uninhabitable or inaccessible during the recovery phase, as buildings and utilities undergo repairs within the larger community. While there is no direct measure of reconstruction progress in the HPS data, there is evidence that single‐family dwellings are repaired and replaced quicker than multi‐family dwellings (see “Dwelling type” in Table 2). However, there is no direct measure of community‐level reconstruction progress.
Place attachment	Place attachment is defined as the strong affective bond people have to their environment (e.g., Hidalgo & Hernández, 2001; Scannell & Gifford, 2010). Place attachment has frequently been cited as a determinant of household return after disasters (e.g., Bonaiuto et al., 2016; Chamlee‐Wright & Storr, 2009; Morrice, 2013). Past research that studied the influence of place attachment on household return typically used proxies, such as tenure duration, hometown status, or neighborhood satisfaction (e.g., Costa, Wang, et al., 2022; Y.‐J. Lee et al., 2017; Nejat & Ghosh, 2016). While this study includes “Tenure status” (see Table 3) as an explanatory variable, other proxies were unavailable in the HPS data.
Social capital	Social capital, or the “resources embedded in one's social networks (…) that can be accessed or mobilized through ties in the networks” (Aldrich, 2011; Lin, 2008), has also been identified as an influential factor in the return decisions of displaced households (e.g., Henry, 2013; Y.‐J. Lee et al., 2017). Types of social capital include bonding (within networks), bridging (between networks), and linking (formal collaboration with institutions; e.g., Aldrich and Meyer, 2015; Nakagawa and Shaw, 2004; Woolcock, 2002). Bonding social capital is partially captured through the “Household size” and “Marital status” variables (see Table 3), representing family and relationships. However, bridging and linking social capital is not explicitly considered an explanatory variable.
Disaster assistance received; Insurance	Insurance and other forms of external assistance were found to be potentially significant for household decisions to rebuild or repair after the 2012 Hurricane Sandy in Staten Island, NY (Nejat & Ghosh, 2016). However, direct information on the assistance received (e.g., from the government, insurance, nonprofits, and family/friends) was unavailable in the HPS data. Disaster assistance moderates the role of household income (see “Household income per family member” in Table 3), and timely disbursement of assistance can enable households to initiate repairs more quickly. In the United States, a major disaster declaration (see “Disaster declarations (2021–2023)” in Table 4) is also required to get access to government disaster assistance through the Federal Emergency Management Agency (FEMA).
Post‐disaster housing reconstruction approach	Post‐disaster housing recovery policies (e.g., targets for the provision and construction of temporary and permanent housing, and establishment of new standards for where and how to build) can influence the nature of population return. In the 20th and 21st centuries, the United States has largely followed a “limited intervention” approach to housing construction after disasters. In this approach, insurance and some forms of limited governmental assistance exist, but it is generally assumed that the real estate market will address housing recovery issues (e.g., Comerio, 1998; Peacock et al., 2018). Additional post‐disaster policies could be implemented at the state or local level, but these data were unavailable in the HPS dataset.

## 3 METHODOLOGY

The records from the HPS are comprised primarily of qualitative (i.e., categorical) attributes, including the displacement duration attribute, which allowed the following responses:

- “Less than 1 week”
- “More than 1 week but less than 1 month”
- “1–6 months”
- “More than 6 months”
- “Never returned home”: Throughout this study, we refer to this class as “Not returned” instead, as the survey does not ask respondents if this is their final decision.

Therefore, different supervised learning algorithms (i.e., inputs and outputs labeled) for classification problems (i.e., qualitative data) were considered (El Naqa & Murphy, 2015). Logistic regression models have significant precedent for use in this research area (e.g., Burton et al., 2019; Kim & Oh, 2014; Nejat & Ghosh, 2016) and are appealing due to their ease of interpretation. However, logistic regression models require the removal of highly correlated input variables and assume a uniform relationship between the input and output variables (Ranganathan et al., 2017). The HPS dataset has several highly correlated variables of interest (e.g., property damage with water shortages after disaster and educational attainment with household income), making logistic regression models less appealing. Decision/classification tree models retain ease of interpretation while better handling correlated features (Speybroeck, 2012). Classification trees also mimic the phenomena (i.e., the household decision‐making process; James et al., 2023) and allow the total number of predictors to be restricted, simplifying implementation within disaster risk analyses. Including numerous new predictors not typically considered within disaster risk models would be impractical, as attaining additional exposure data (such as household demographic attributes) and capturing their correlation with physical attributes is complex (e.g., Menteşe et al., 2023). Random forests are ensemble models where multiple decision trees are used with the chosen class determined by majority rule, improving the performance of individual trees at the cost of interpretability (James et al., 2023) and sharing many of the advantages of decision trees. Naïve Bayes models are computationally efficient and well suited to categorical data (Webb et al., 2010). However, Naïve Bayes models assume all features are independent, which seems inappropriate for the HPS data. Support vector machines and K Nearest Neighbor models were also considered but had comparable or inferior performance without any clear advantages for the study. Therefore, classification trees and random forests were deemed most suitable, given their improved handling of correlated features and ability to mimic the household decision‐making process. Although both classification trees and random forests were deemed most suitable for this particular study, the results for alternative machine learning algorithms are summarized in Appendix B and included as a supplement in the code repository.

**FIGURE 4:**
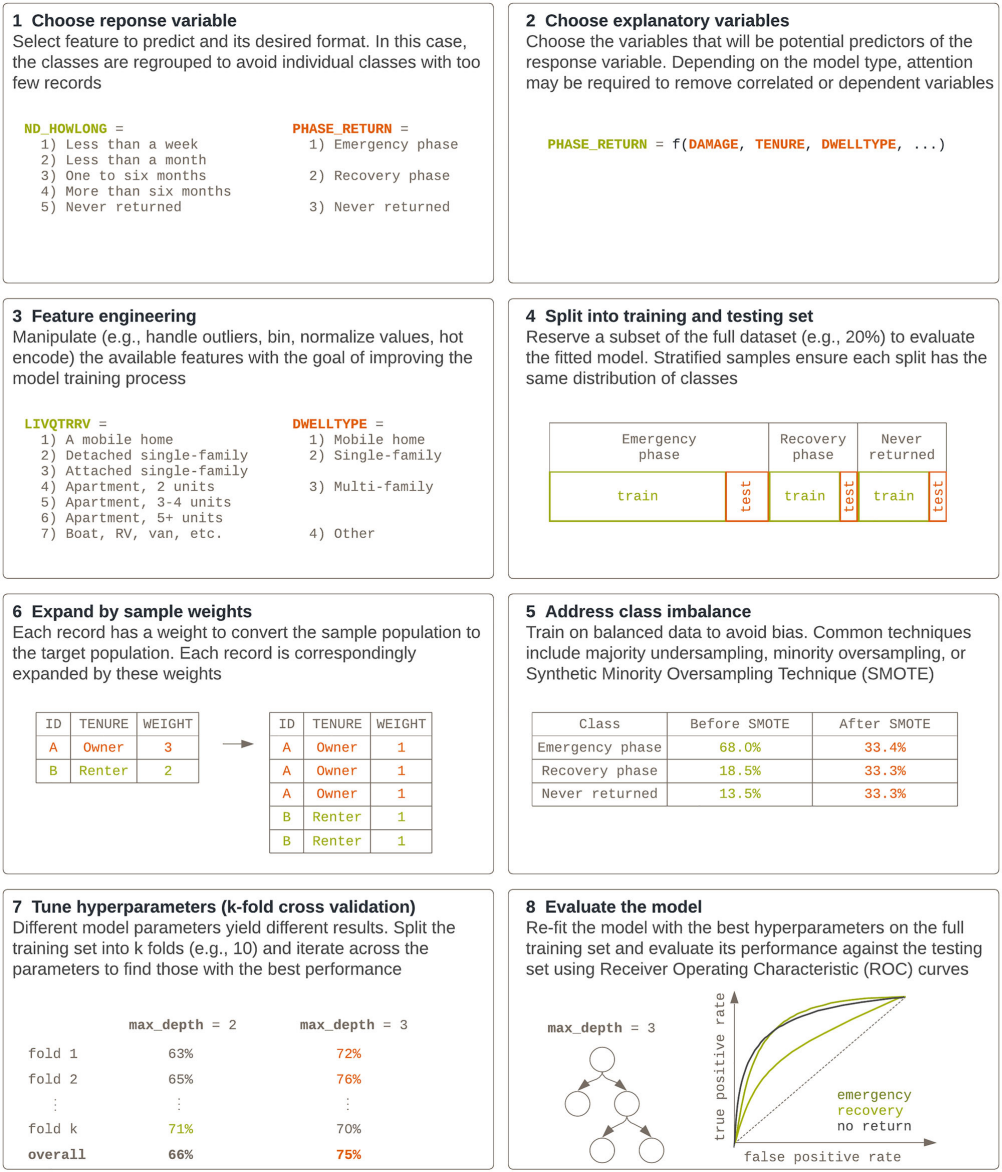
High‐level overview of the methodology used to fit machine learning algorithms to the Household Pulse Survey (HPS) data. Some variable names per the HPS are referenced, such as ND_HOWLONG for the duration of displacement and LIVQTRRV for the living quarters.

An overview of the methodology for model fitting is shown in Figure 4. Each step is described further in the following subsections. The code to reproduce the models or adjust specific modeling assumptions is available at https://github.com/nicolepaul/hps‐displacement.

### 3.1 Choosing the response variable

Household displacement duration is the response variable, which in the original format consisted of five potential categories: less than 1 week, more than 1 week but less than 1 month, between 1 and 6 months, over 6 months, and not returned. However, initial model fitting and evaluation yielded poor performance for two minority classes: between 1 and 6 months and over 6 months (see performance in Appendix B). Therefore, the response variable was re‐binned to produce fewer classes with larger sample sizes: emergency phase displacement (displacement durations less than 1 month), recovery phase displacement (displacement durations beyond 1 month), and not returned (potentially permanent relocation). The number of samples in each class is shown in Table 6. Despite the rebinning, class imbalance remained and was addressed as described in section 3.6.

**TABLE 6:** Selection of the response variable for displacement durations using the Household Pulse Survey (HPS) dataset.

Response variable	Sample size (proportion^a^)	Original class	Sample size (proportion^a^)
Emergency phase displacement	7536 (65.6%)	Less than 1 week	5055 (42.5%)
		Less than 1 month	2481 (23.1%)
Recovery phase displacement	2229 (20.3%)	1–6 months	1363 (12.2%)
		More than 6 months	866 (8.1%)
Not returned	1463 (14.1%)	Not returned	1463 (14.1%)

^a^ The proportions are calculated after applying the household weights included within the public use files (PUFs). These household weights are provided to adjust for nonresponse bias, allowing us to map from the sample population (i.e., those that responded to the survey) to the target population (i.e., all households in the United States, excluding territories). Therefore, the proportions may not correspond exactly to the sample size values.

Importantly, the survey did not inquire whether individuals in the “Not returned” class had made a final decision or how much time had passed since their initial displacement. Therefore, there is likely some overlap between the “Not returned” class and the other two classes. However, the descriptive statistics and model fitting results yield distinct trends for the “Not returned” class, so it was kept separate from the other two. We believe that this class indicates a high potential for permanent relocation, but we acknowledge the data limitation.

### 3.2 Choosing the explanatory variables

The explanatory variables were selected primarily based on a literature review of household displacement and the determinants of return, as described in section 2.3. Appendix A provides a data dictionary defining each data type, range of values, and source.

### 3.3 Feature engineering

Household attribute data from the HPS records were primarily categorical (ordinal or nominal). The few continuous or discrete variables (i.e., age and household size) were binned to become ordinal, where the bin edges were selected to achieve a relatively consistent frequency across each bin (i.e., to avoid classes with a small minority of samples). Some of the nominal and ordinal data were also binned more coarsely for simplicity and to avoid having multiple classes with limited representation (e.g., all multi‐family dwelling types were grouped into a single category rather than being differentiated by the number of units). Lastly, household income was normalized by the number of household members.

Area attributes (listed in Table 4) were appended to the household records based on the respective geographic units of each respondent. Unfortunately, the spatial resolution of geographic data for each household record in the HPS PUFs is coarse. Each record indicates the respondent's state and, if relevant, which of the top 15 Metropolitan Statistical Areas (MSA) the respondent resides in. Area attributes from the United States Census Bureau were appended to each record based on the corresponding MSA (if relevant) or state (otherwise). Additionally, the number of major disaster declarations within the survey period (2021–2023) for each state was appended to each record using open data from the Federal Emergency Management Agency (OpenFEMA, available at https://www.fema.gov/about/openfema/data‐sets).

### 3.4 Splitting into testing and training datasets

After the feature engineering step, the dataset was split into training and testing sets to ensure the model captures the phenomena without overfitting. A general rule of thumb is to use 70%–80% of the dataset for training and 20%–30% for testing (e.g., Gholamy, Kreinovich, and Kosheleva, 2018). Given the relatively small sample size of the dataset, 20% was reserved for the testing set to maximize the number of samples used for training. Stratified samples were taken to ensure that the proportion of each class (i.e., emergency phase displacement, recovery phase displacement, and not returned) in the training and testing sets is equal to that of the entire dataset. This split occurs randomly rather than chronologically to avoid potential spatiotemporal correlations across different disaster events reported over time. Notably, the HPS asks about experiences of disaster displacement over the past 12 months, implying a moving timeframe of 12 months for each response.

### 3.5 Expanding by household weights

As with many household surveys, the HPS dataset includes individual and household weights to map from the sample population (i.e., those that responded to the survey) to the target population (i.e., all households in the United States). These weights correct for nonresponse bias and other factors that could reduce the sample's representativeness. According to the HPS PUFs, the household weights for the displaced households ranged from 47.66 to 66,280.97. If these weights are not applied, model training and evaluation would be representative of the sample population and not the target population. Therefore, each record was expanded in proportion to the household weights provided by the HPS PUFs. To avoid an excessive data sample size, all household weights were divided by the smallest value (47.66) and rounded to the nearest integer. In this manner, each response was duplicated between 1 and 1326 times. Since expanding by the household weights effectively duplicates each response, this step occurred after splitting the dataset into the training and testing sets. Otherwise, several responses within the testing set would be identical to those used in training as an artifact of the methodological choice rather than a product of good predictive performance.

### 3.6 Addressing class imbalance

As was identified in Table 6, most households returned in the emergency phase (less than 1 month after the disaster). Models fit on imbalanced training data such as these are prone to poor performance (e.g., biasing toward the majority classes), so class imbalance in the HPS dataset was addressed. Standard methods of addressing class imbalance include majority undersampling, minority oversampling, and creating synthetic samples. For this study, the Synthetic Minority Oversampling Technique, or SMOTE, was used. SMOTE is an oversampling technique where synthetic samples are created for the minority class(es) until the class balance is achieved (Chawla et al., 2002). SMOTE was selected as it has been employed successfully for related studies, such as quantifying place attachment (Costa, Wang, et al., 2022) and post‐earthquake business recovery (Costa & Baker, 2021). This process was only applied to the training set to ensure the purity of the testing set. As a result, the testing set retains the class imbalance inherent to the original dataset. Consequently, individual class scores and macro‐averages were emphasized during model evaluation.

### 3.7 Tuning hyperparameters

Each machine learning algorithm supports a variety of hyperparameters set before model training (i.e., they are not learned from the data). Hyperparameters can impact model performance and behavior, but determining the optimal values is not trivial and often involves an iterative search process. This study uses a grid search approach with 10‐fold cross‐validation to identify the ideal hyperparameters. The key scoring metrics used to evaluate the models were macro‐averages of the F1 score (a metric signifying balanced precision and recall; Opitz and Burst, 2021) and the Area Under Curve (AUC; see section 3.8 for an elaboration). Consistency (i.e., the standard deviation across all 10 folds) and model complexity (i.e., the number of predictors required) were also considered alongside the average evaluation scores to select the ideal hyperparameters.

After identifying the ideal hyperparameter values, the model was refit using the entire training set before proceeding to the final model evaluation step. A summary of the considered and selected hyperparameter values for the full models (i.e., including both physical and socioeconomic factors) is shown in Tables A4 and A5 in Appendix B. For the classification tree model (TreeP&S), the considered hyperparameter values were designed to prevent overfitting and targeted a simple model to ensure practicality within disaster risk analyses. The selected classification tree model parameters yielded a slightly lower average score than the top‐scoring model but had higher consistency and lower complexity. The hyperparameter settings and scores for the top 10 ranking models are also reported in Appendix B.

### 3.8 Evaluating the model

The testing set was used to evaluate the final models by comparing the model predictions against the actual values. Since the testing set is imbalanced, metrics affected by the number of samples in each class were avoided (e.g., overall accuracy). That is, metrics were calculated for each class separately, and the overall performance was evaluated using the macro‐average across all classes.

Each model's predictive power was evaluated using receiver operating characteristic (ROC) curves and the area under the ROC curve (AUC; Carter et al., 2016). ROC curves provide a graphical view of the performance of a binary classifier; however, the proposed models use three classes rather than two. Thus, the ROC curve for each class was calculated using a one‐versus‐rest approach (i.e., the selected class equals 1, whereas the other classes equal 0). The ideal result is a ROC curve that follows the graph's upper left corner. Accordingly, an AUC of 1 would represent a perfect model. The rule of thumb in medical diagnostic testing is that AUC values above 0.7, 0.8, and 0.9 have fair, good, and excellent performance, respectively (e.g., Muller et al., 2005; Nahm, 2022). However, appropriate benchmarks for models of human behavior are not well established.

Confusion matrices are also presented to help understand the distribution of errors. A confusion matrix cross‐tabulates the predicted classifications of the model versus the actual classifications in the testing set. The diagonal cells represent the correct predictions, whereas the off‐diagonal cells represent the erroneous predictions. Since the testing set is imbalanced and to ease interpretation, the value in each cell was normalized.

## 4 PREDICTIVE MODELS AT THE HOUSEHOLD LEVEL

This section documents the performance of and takeaways from the proposed classification tree and random forest models. The classification tree models are proposed for practical integration into disaster risk models, as they require relatively few predictors. The random forest model, in contrast, incorporates all explanatory variables as predictors and thus enables a broader understanding of the drivers of different displacement outcomes. Appendix B summarizes the performance across all proposed and alternative models, including different machine learning algorithms and hyperparameter settings. Additionally, the code for the proposed and alternative models is available in the code repository.

### 4.1 Classification trees: Simple predictive models for displacement duration

Since classification tree models were deemed the easiest to integrate within disaster risk analyses, two model variants were considered. The first considers all explanatory variables (i.e., both physical and socioeconomic factors), whereas the second only considers physical factors that are traditionally modeled in disaster risk analysis (i.e., representative of the standard practice).

**FIGURE 5:**
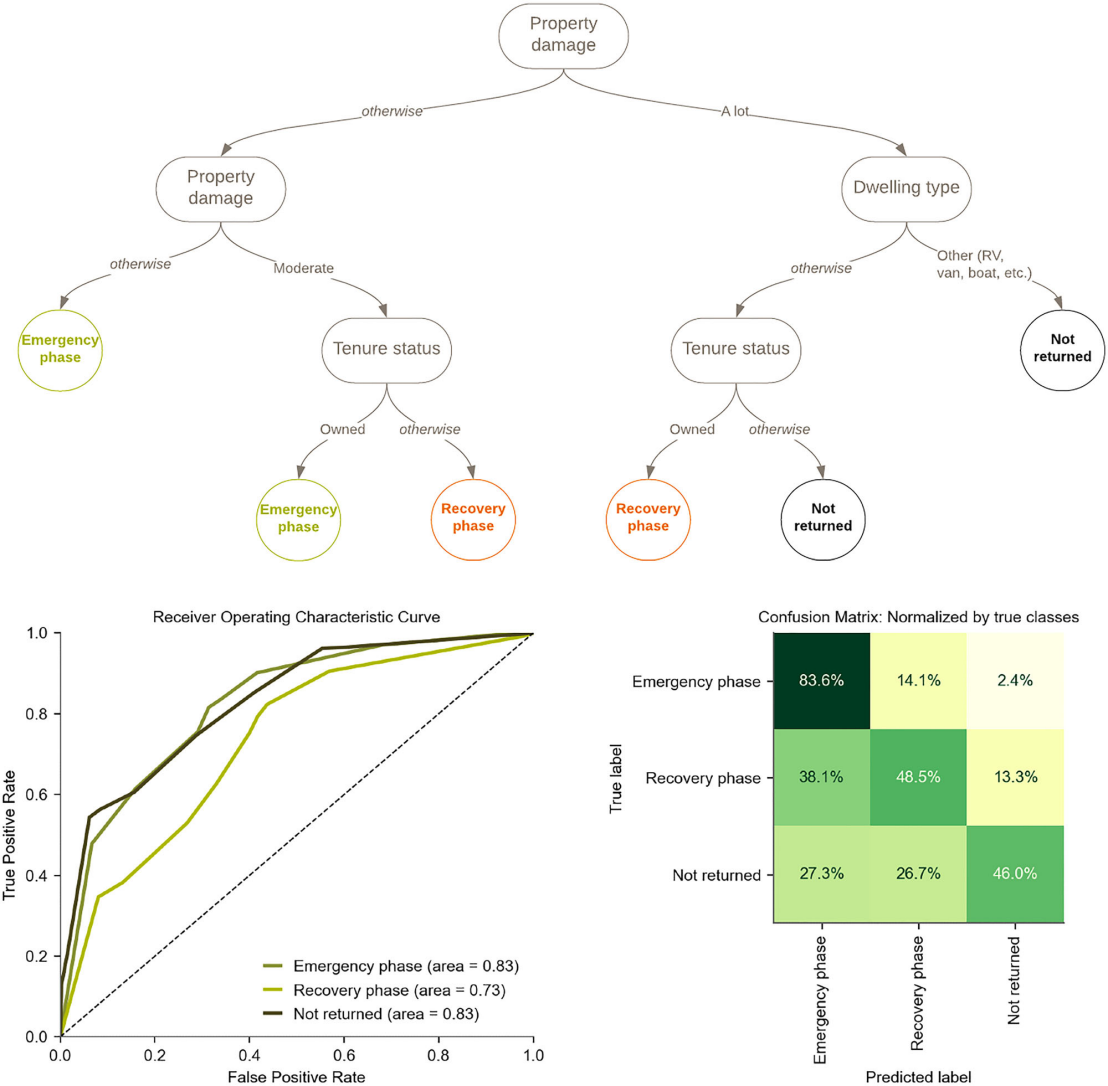
(Top) TreeP&S: The classification tree model for displacement durations considering physical and socioeconomic factors, and (Bottom) model evaluation using the receiver operating classifier (ROC) curves and the confusion matrix.

The final classification model considering all explanatory variables is shown in Figure 5 and is referred to as “TreeP&S.” Although all explanatory variables are considered in model fitting, the resulting model only requires three predictors: the level of property damage, dwelling type, and tenure status. The splits’ order indicates the relative importance level of each explanatory variable; thus, property damage is the primary driver of household displacement durations, a result that is consistent with disaster literature (e.g., Burton et al., 2019; Comerio, 1997; Cong et al., 2018; Mayer et al., 2020; Scheele et al., 2020; Smith & McCarty, 1996). The first split by damage separates households that self‐rated their level of property damage in the highest category (“a lot”) versus all other households. The subsequent splits are based on property damage (again) and dwelling type. This second split by damage separates households that self‐rated their level of property damage in the second highest category (“moderate”) versus households that chose the lowest two categories (“none” and “a little”). The dwelling type split separates those who live in less common living quarters (e.g., recreational vehicles, vans, and boats) from those who live in more common living quarters (i.e., single‐family homes, multi‐family homes, and mobile homes). This split by dwelling type highlights the increased mobility of those who may have either failed to enter or otherwise opted out of the conventional housing market. The third and final splits are both based on tenure status. In both splits by tenure status, owners are more likely than nonowners to return during the emergency phase than the recovery phase and are also more likely to return than not return. These splits by tenure verify observations from past disaster events: the quicker return of homeowners may be partially explained by higher resource availability (e.g., personal savings and access to insurance and post‐disaster assistance; J. Y. Lee & Van Zandt, 2019), whereas the conscious decision to return could be partially explained by higher levels of place attachment (Binder et al., 2015; Henry, 2013) or mortgage obligations (Elliott & Pais, 2006).

This model is relatively simple yet nearly as good as the more complex random forest model in predicting displacement duration (see section 4.2). There is the least confusion in identifying the emergency phase displacement class, with 83.6% of those samples correctly identified in the testing set. As desired, the diagonal of the confusion matrix exhibits the highest percentages, signifying that samples in the testing set were usually classified correctly. At the same time, there is some notable confusion across the recovery phase displacement and not returned classes. This result may be partly due to the complexity and variability of household decisions to return or resettle as they experience more prolonged displacement durations. This could also be partially attributed to the misclassification of households in the emergency phase and recovery phase of displacement into the “Not returned” class, as the survey does not ask respondents whether their “Not returned” status is a final decision. Overall, the classification tree model considering all explanatory variables performs well, and the driving factors seem consistent with the literature review.

**FIGURE 6:**
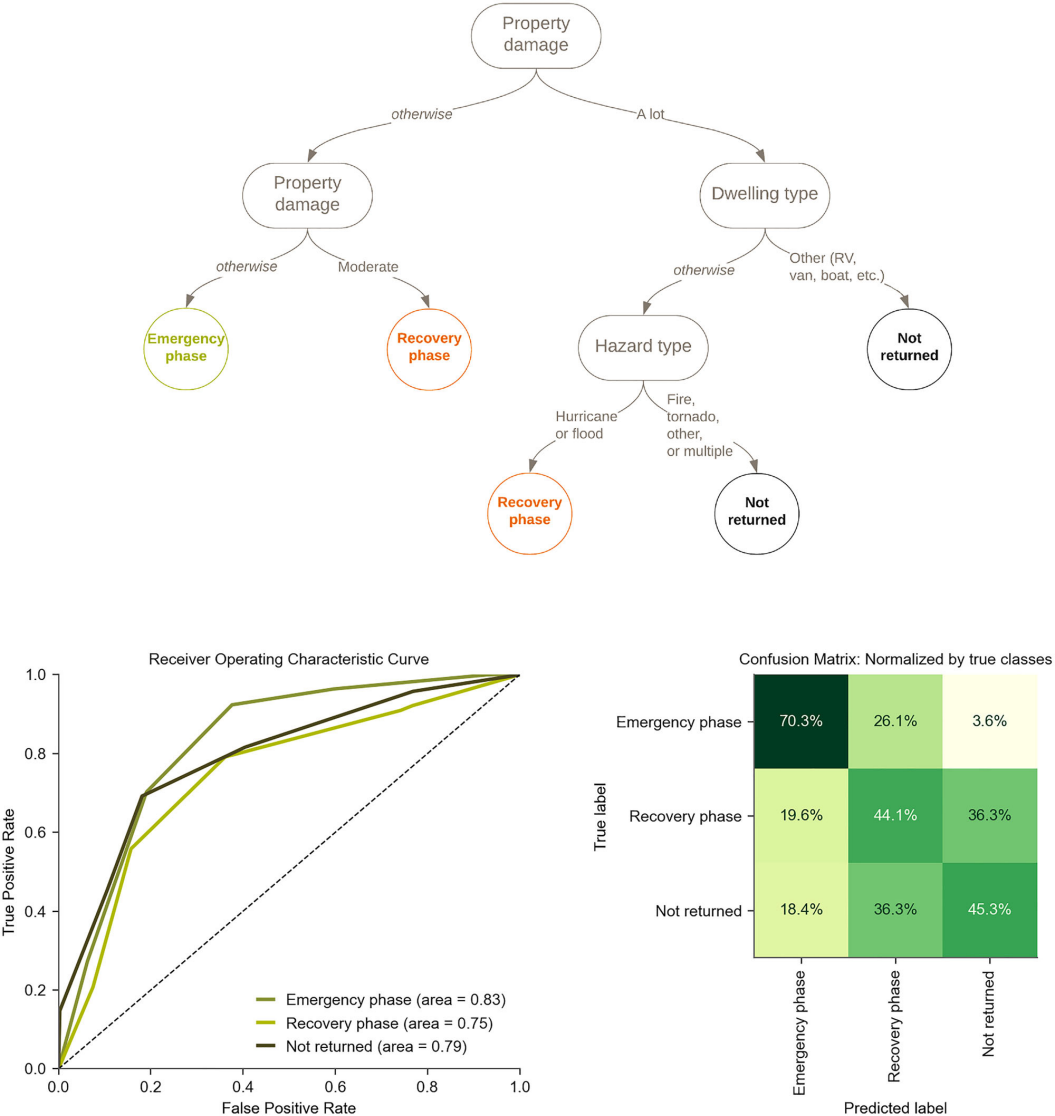
(Top) TreeP: The classification tree model for displacement durations considering only physical factors, and (Bottom) model evaluation using the receiver operating classifier (ROC) curves and the confusion matrix.

The results for the classification tree model using only physical factors are shown in Figure 6. This model is referred to as “TreeP.” The primary split is based on property damage, the same as the model with socioeconomic factors included. The second level of splits is also identical to the model with socioeconomic factors—one split by property damage (again) and the other by dwelling type. The third and final split is distinct from the TreeP&S model: by hazard type. Residents who experienced a fire, tornado, or multiple hazard types are assigned to “not returned,” while residents who experienced a hurricane or flood are assigned to “recovery phase displacement.” However, there is notable confusion between the ‘‘not returned and “recovery phase” displacement classes, suggesting that this model should be treated as a binary classifier instead (i.e., with the recovery phase displacement and not returned classes grouped).

### 4.2 Random forest: Inferring factors that influence displacement durations

A random forest model considering both physical and socioeconomic factors was also fit, referred to as “ForestP&S.” Random forests offer the opportunity to improve model performance and quantify the contribution of all considered explanatory variables, but at the cost of adding complexity. Recent methods in XAI, such as Shapley Additive explanation (SHAP) values, have been proposed to interpret the predictions of such complex machine learning models (Lundberg & Lee, 2017) and can be efficiently calculated for random forests using the TreeExplainer method (Lundberg et al., 2020). SHAP values are based on cooperative game theory and describe each explanatory variable's marginal contribution to a given model prediction. In other words, the final probability of a given class is equal to the baseline probability plus the sum of the SHAP values across all explanatory variable values. Since the model was fit on balanced data, the baseline probability for each class (e.g., emergency phase displacement) is uniform (i.e., 33%). A SHAP value of 5% for a given sample indicates an increase in the probability of that class by 5%, whereas a SHAP value of −5% decreases the prediction of that class by 5%. The SHAP values are calculated for each data sample (i.e., each household), for each explanatory variable, and for each predicted class.

**FIGURE 7:**
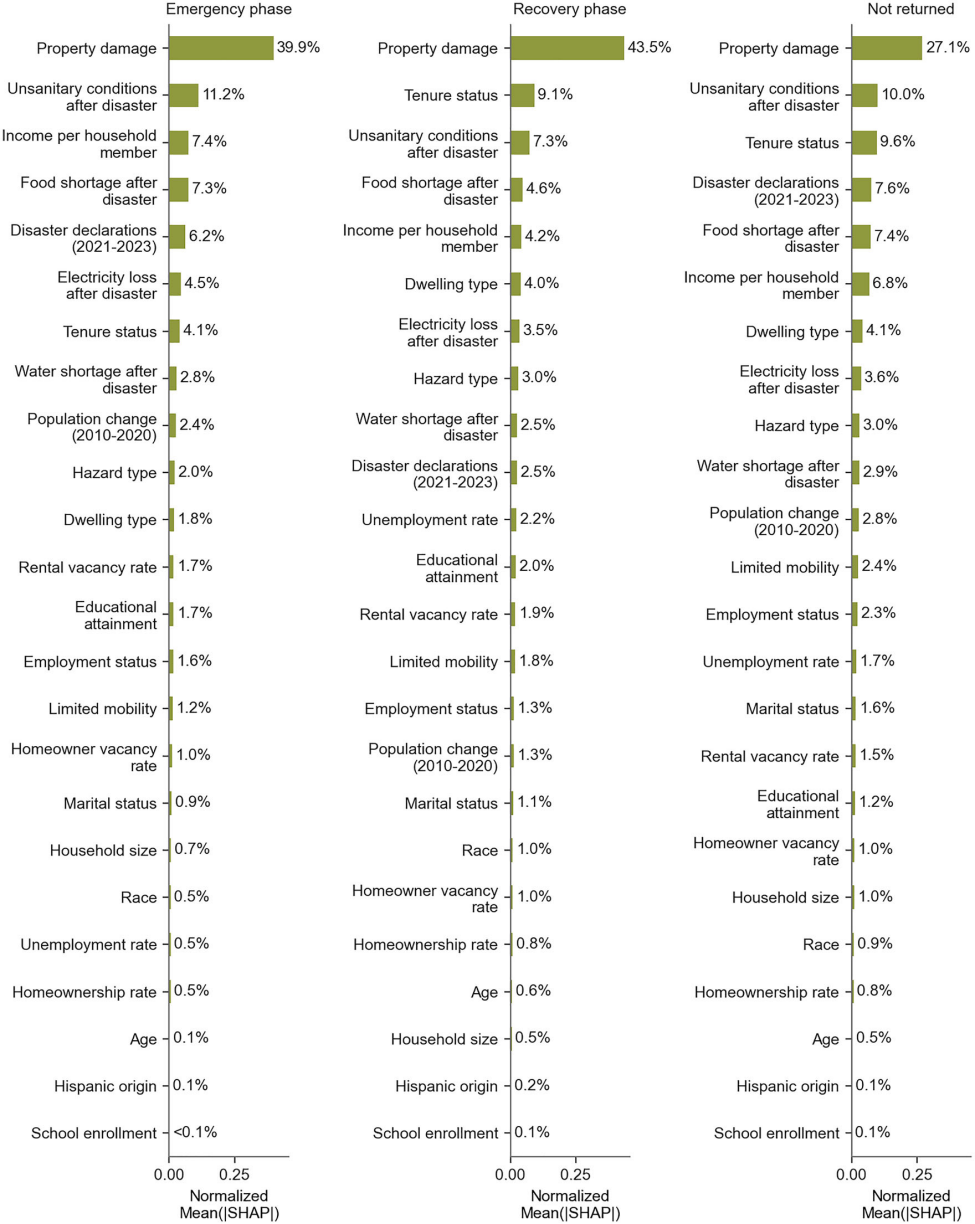
Global explanation of ForestP&S (the random forest model considering both physical and socioeconomic factors) for each predicted class: emergency phase displacement, recovery phase displacement, and not returned. SHAP, Shapley additive explanation.

Figure 7 provides a global explanation of the random forest model by averaging the absolute SHAP values across all households for each predicted class and explanatory variable. To ease interpretation, the aggregate SHAP values for each class are normalized such that they sum to 100%, and the order of variables on the y‐axis is sorted accordingly. From these results, property damage is highlighted as the primary driver of all predicted return outcomes, a result that is consistent with the tree models and with the literature review. On average, property damage explains roughly 40% of return predictions (either in the emergency phase or the recovery phase) but less than 30% of not‐returned predictions. After property damage, the explanatory variable ranking varies depending on the predicted class. The top five factors for the emergency phase class are nearly all physical factors: property damage, unsanitary conditions after the disaster, food shortage after the disaster, and the number of disaster declarations in the surveyed period. The only exception is the income level per household member, which ranks as the number three contributor to emergency phase displacement predictions. In contrast, the recovery phase class and the not returned class feature two socioeconomic factors in their top five rankings: tenure status and the income per household member. Across all classes, the features with the least global importance are Hispanic origin and school enrollment.

**FIGURE 8:**
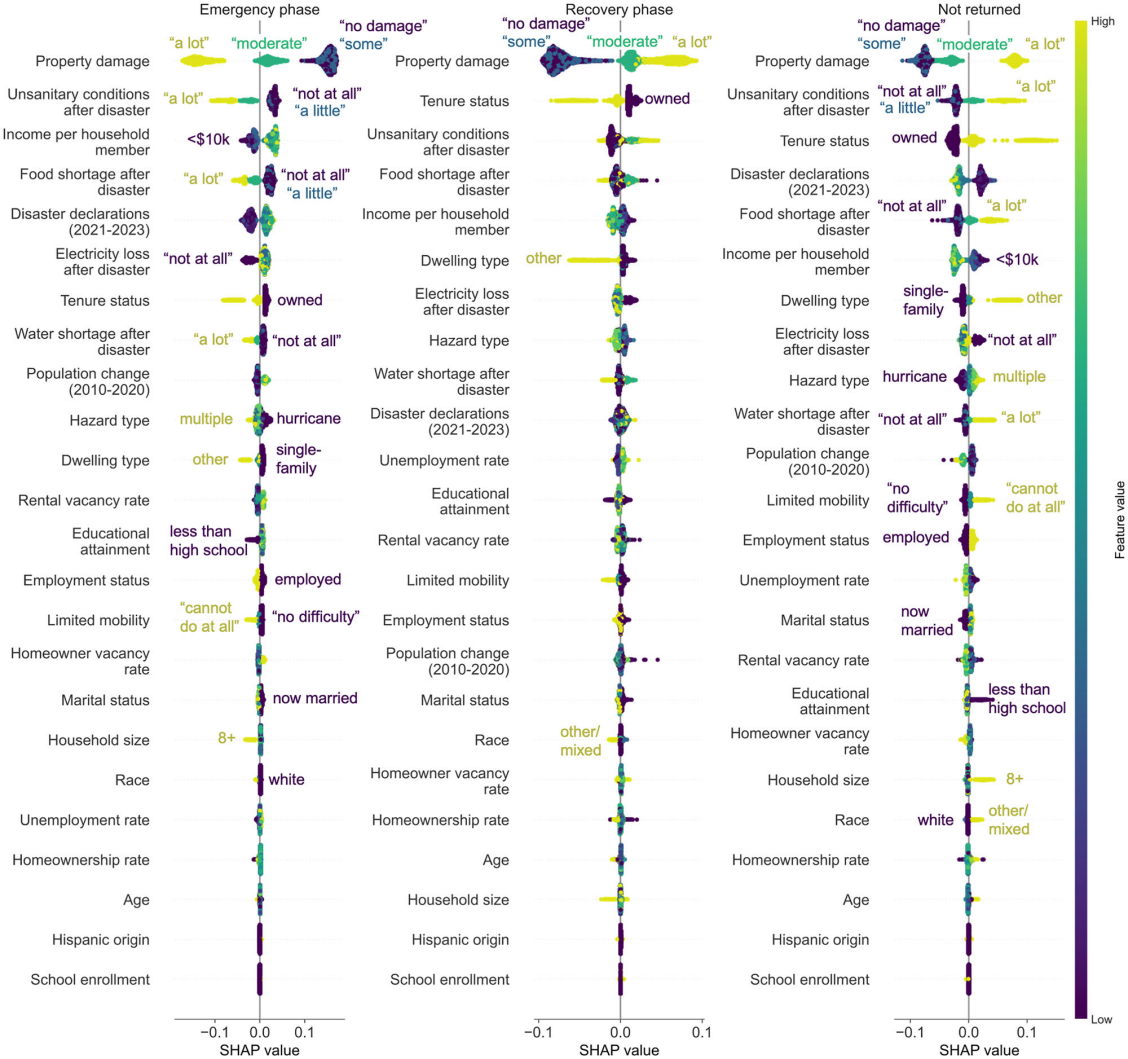
Global and local explanations of ForestP&S (the random forest model considering both physical and socioeconomic factors). A beeswarm plot is shown for each predicted class: emergency phase displacement, recovery phase displacement, and not returned. SHAP, Shapley additive explanation.

Both global and local explanations can be visualized together using a beeswarm plot, as shown in Figure 8. Here, the SHAP value is plotted along the x‐axis for each household: values to the left of the center line indicate that a household is less likely to be predicted in that class, whereas values to the right of the center line indicate that a household is more likely to be predicted in that class. The location of each point indicates the local impact (i.e., for that sample household), whereas the order of variables on the y‐axis is based on the global impact (i.e., the average absolute SHAP value across all samples, same as in Figure 7). The color of each marker is based on the feature values, where lighter colors indicate higher values and darker colors indicate lower values. Annotations are included for the ordinal and nominal features that demonstrate clear trends between the feature value and model impact. Notably, some features with lower global importance (e.g., household size or physical mobility for the not returned class) have significant local importance. This is particularly true for many edge cases of feature values (e.g., household size of 8+, dwelling type in the “other” category, and educational attainment level of “less than high school”).

Beyond the importance of property damage across all displacement outcomes, some other clear signals emerge in Figure 8. There is a clear distinction by tenure status, whereby homeowners are more likely to return, regardless of whether in the emergency phase or recovery phase. This propensity for homeowners to return has long been reported in past disaster literature (e.g., Cong et al., 2018; Elliott & Pais, 2006; Kim & Oh, 2014; Y.‐J. Lee et al., 2017; Mayer et al., 2020). Meanwhile, households in the lowest two income brackets (<$10,000 per member and <$20,000 per member) are more likely to return in the recovery phase than in the emergency phase and are also more likely to not return as compared with other displaced households. Previous disaster literature also often cites recovery challenges for low‐income households, such as having less access to post‐disaster assistance and fewer personal resources to cover moving costs, repair costs, or rent increases (e.g., Fothergill & Peek, 2004; Pardee, 2012; Peacock et al., 2018). Similarly, the increased mobility of households with low educational attainment has also been observed in past disasters (e.g., Mayer et al., 2020; Morrow‐Jones & Morrow‐Jones, 1991). The results also signify some discrepancies by hazard type: households that reported displacement due to hurricanes tended to return faster, whereas those who reported displacement due to multiple hazards were more likely to not return.

**FIGURE 9:**
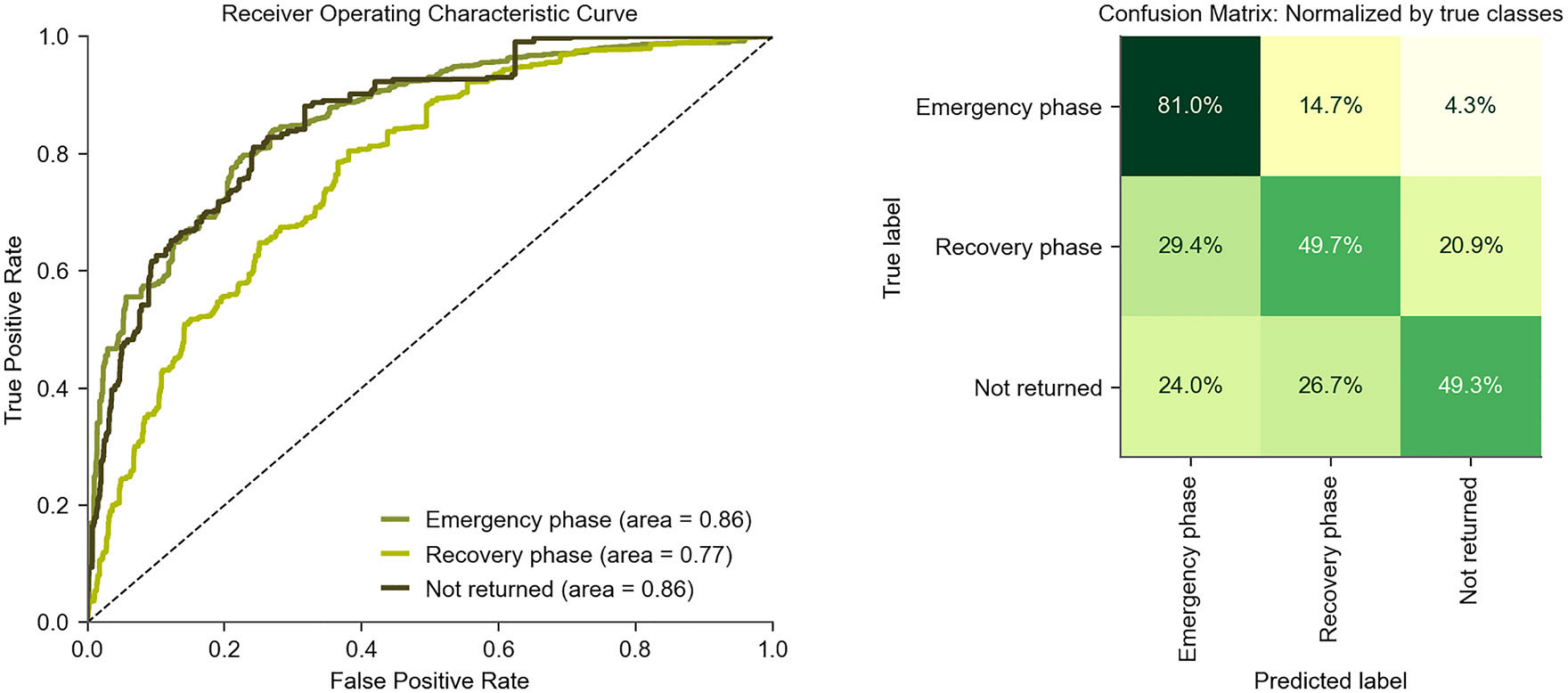
The receiver operating characteristic (ROC) curves and the confusion matrix for ForestP&S (the random forest model considering both physical and socioeconomic factors).

These explanations are only as strong as the model's predictive power; therefore, confidence in the findings should be moderated by the performance of each class, which is shown in Figure 9. The ForestP&S model predicts all three displacement classes better than the classification tree models, indicating that a broader range of factors influences outcomes than just the three within the TreeP&S model (i.e., property damage, dwelling type, and tenure status). Although the ForestP&S model performs slightly better, it requires the inclusion of several explanatory variables beyond the classification tree models. It would, therefore, be challenging to integrate into disaster risk analyses.

## 5 APPLICATION AT THE COMMUNITY LEVEL

A hurricane scenario analysis was performed to demonstrate the predictive models for household displacement durations at the community level, considering both classification tree model variants: TreeP&S (with socioeconomic factors included) and TreeP (considering only physical factors). The scenario analysis was performed with open tools and data developed by the Natural Hazards Engineering Research Infrastructure's Computational Modeling and Simulation Center (NHERI SimCenter; Deierlein & Adam Zsarnóczay, 2021). This includes the Atlantic County, New Jersey, testbed (Kijewski‐Correa et al., 2022) and the Regional Resilience Determination tool (R2D; McKenna et al., 2024). NHERI SimCenter's regional testbeds include hazard and exposure input data, which can be used to conduct regional risk analyses in R2D.

**FIGURE 10:**
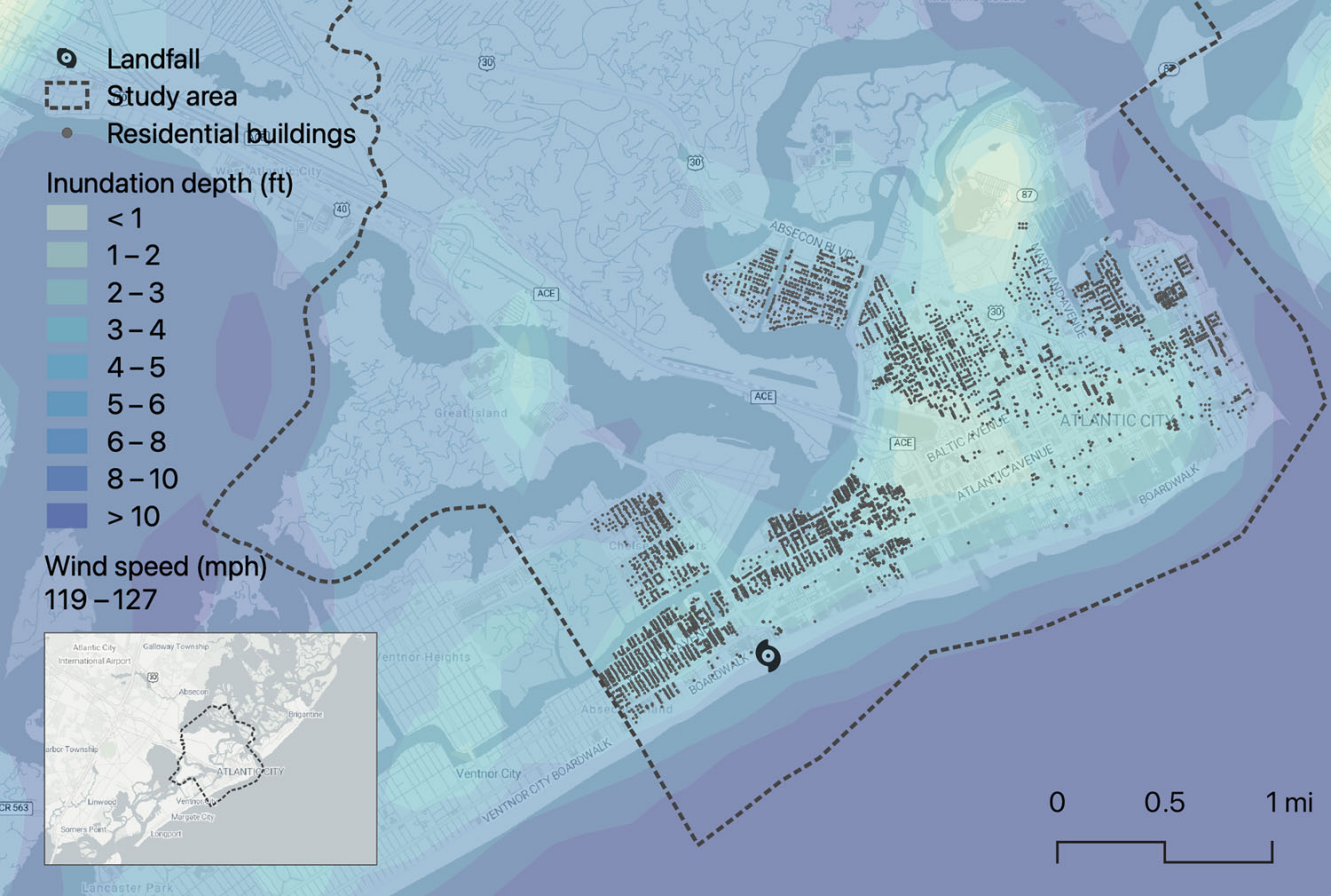
Map of the scenario risk model: A Category 3 hurricane with landfall in Atlantic City, NJ.

Wind speeds and inundation depths were based on the synthetic storm scenarios calculated by the Storm Hazard Projection (SHP) Tool developed in the NJcoast project (Kijewski‐Correa et al., 2020). The Category 3 storm was selected since the wind speeds were similar to the design wind speed in Atlantic City, NJ (ASCE, 2022). However, about 85% of the residential building inventory in Atlantic City was constructed before the adoption of building codes in New Jersey (1970s) and 90% before the implementation of modern hurricane design regulations (2000s). All residential buildings in Atlantic City, NJ, were included in the model, where the tenure status was inferred based on fuzzy string matching between the property address and the owner's address listed on the county's tax assessor records (NJOGIS, 2023). If the property address was highly similar to the owner's address (i.e., a simple ratio over 80% or a partial ratio over 85% using the Levenshtein distance; Navarro, 2001), the property was assumed to be owner‐occupied. Otherwise, the property was assumed to be renter‐occupied. This approach neglects vacant or seasonal properties common in the area but was deemed sufficient for the sensitivity study. The appropriate HAZUS wind and flood fragility curves (FEMA, 2003) for each building were assigned based on rulesets that consider the year built relative to the adoption of hurricane construction standards and the roof type (Angeles & Kijewski‐Correa, 2023) and dwelling type. A map of the study area is shown in Figure 10.

Using R2D, 100 realizations of damage states were sampled for each building. For each realization, the building damage states were mapped to a corresponding displacement duration for each household within the building using the proposed classification tree models. It was assumed that households in buildings that were extensively or completely damaged would respond “a lot” to the HPS question regarding the level of property damage experienced. The small number of realizations was deemed appropriate since the hazard characterization comprised a single set of values per intensity measure (i.e., no uncertainty was modeled) and because the coefficient of variation was less than 5% for each displacement outcome. Accordingly, only mean impact estimates are presented in Table 7 (for damage counts) and Table 8 (for displaced household estimates). For this Category 3 scenario with landfall in Atlantic City, NJ, the wind speeds are almost double what was reported during Superstorm Sandy. Combined with the low levels of modern hurricane‐resistant residential construction in the area, the estimated level of damage is significant.

**TABLE 7:** The mean number of Atlantic City, NJ, residential buildings in each damage state and the assumed Household Pulse Survey (HPS) property damage level.

Damage state	Assumed HPS property damage response	Number of residential buildings
None	“No damage”	1154
Slight	“Some damage”	534
Moderate	“Moderate amount of damage”	165
Extensive	“A lot of damage”	476
Complete		1692

**TABLE 8:** Hurricane scenario mean impact results for the percentage of Atlantic City, NJ, households in each displacement class using two classification tree models for household displacement durations: With socioeconomic factors and without (i.e., physical factors only).

Displacement class	TreeP&S model (with socioeconomic factors)	TreeP model (physical factors only)	HPS dataset (hurricane and flood)	HPS dataset (hurricane only)
Emergency phase (<1 month)	44.5%	42.8%	48.0%	73.6%
Recovery phase (>1 month)	28.4%	57.2%	29.7%	17.9%
Not returned	27.1%		22.3%	8.5%

*Note*: For reference, the descriptive statistics for all Household Pulse Survey (HPS) households are also provided.

Both displacement duration models yield consistent estimates for the emergency phase class, indicating that 42.8%–44.5% of households return within 1 month. However, whereas the TreeP&S model uses housing tenure to distinguish recovery phase displacement from not returned at the highest level of damage, the TreeP model cannot distinguish between the recovery phase displacement and not returned classes for a given hazard type. This result signifies that some socioeconomic factors require consideration to distinguish which households are displaced into the recovery phase versus those that do not return, particularly for urban areas (as they tend to have lower homeownership rates) and for highly damaging disaster events. For the TreeP model, depending on how a respondent is likely to report this hazard event, all dwellings in the highest damage state are either assigned to recovery phase displacement (if reporting “hurricane” only) or to not returned (if reporting both “hurricane” and “flood”). Given the scale of wind speeds and inundation depths estimated for this scenario, we expect most respondents to report both “hurricane” and “flood.” This distinction in the TreeP model and in the differences between the descriptive statistics from the HPS dataset highlights the importance of considering the cumulative impact of concurrent or consecutive hazards.

## 6 DISCUSSION AND LIMITATIONS

While the HPS dataset offers an unprecedented lens into household experiences of displacement in recent American disasters, it has some limitations. One major limitation is the interpretation of the “Not returned” class: the survey does not ask these respondents whether or not this is a final decision, nor does it inquire about the time of the disaster event relative to the time of the survey. This makes it challenging to ascertain what proportion of the “Not returned” class has been misclassified, which complicates the interpretation of what drives permanent relocation versus temporary displacement. Another limitation is the subjectiveness of household responses: for example, self‐reported damage levels are likely inconsistent across different households and do not necessarily correspond to established damage scales used in disaster risk analysis (e.g., the European Macroseismic Scale, EMS‐98; Grünthal, 1998). Further, some key factors of interest to policy evaluation, such as insurance or assistance received, are missing, as well as other potentially relevant factors for permanent relocation (e.g., place attachment). The spatial resolution in the HPS is also relatively coarse (i.e., the state or, if relevant, the top 15 MSA). This prevents the robust inclusion of local area attributes such as vacancy rates, long‐term population trends, and the number of disaster declarations. Lastly, the transferability of findings between the dataset's geographic coverage (all 50 states plus the District of Columbia within the United States) to other contexts is unknown.

Model evaluation was possible at the household level by reserving a testing set. Relative to the displacement in the emergency phase and not returned classes, the displacement in the recovery phase class was the most difficult to predict. This could be partly due to a lack of key explanatory variables for this class (e.g., insurance) or the subjectiveness of household responses on key factors (e.g., regarding the property damage level). However, there is also likely some variability from household to household on whether or not to return when experiencing protracted displacement, even if other factors (e.g., damage level, household size, and educational attainment) are identical. A perfectly predictive model is likely unrealistic for this type of complex human behavior. The household‐level model evaluation is informative, but the envisioned use case for the models is for community‐level disaster risk assessment. Ideally, a validation study would be performed for a recent disaster in the United States that occurred outside the surveyed period. This external validation study would require estimates of population displacement durations after the disaster event (e.g., via household surveys or mobile location data; Paul et al., 2024) and disaggregated housing damage statistics (i.e., damage level by dwelling type and tenure status). However, longitudinal displacement estimates and disaggregated damage data are rarely available for past disasters.

## 7 CONCLUSIONS

This study proposes predictive models for the duration of household displacement after disasters, calibrated using survey data from the United States Household Pulse Survey (HPS). Unlike most disaster risk models, this model aims to capture household displacement's duration using information on physical damage and socioeconomic characteristics as predictor variables. Furthermore, the geographic and temporal scope of the survey allows for a broader understanding of displacement duration and return drivers beyond individual case studies. The classification tree model proposed herein could be immediately integrated into any disaster risk model with data on property damage, dwelling type, and tenure status. These estimates of displacement duration and return could be considered alongside other standard risk metrics (e.g., direct economic loss) to capture the human toll of disasters more holistically (e.g., Galasso et al., 2021; Markhvida et al., 2020).

Survey responses from 11,715 households that experienced disaster displacement were used to fit predictive models for household displacement in three classes: emergency phase displacement (less than 1 month), recovery phase displacement (more than 1 month), and not returned (potentially permanent relocation). Both classification tree and random forest models were proposed: TreeP&S, TreeP, and ForestP&S. The classification tree models were fit primarily for straightforward integration within disaster risk analyses, using a minimal number of predictors. In contrast, the random forest model improves performance and allows us to infer the impact of all considered explanatory variables on predictions of each of the three classes. Consistent with prior disaster literature, property damage is the primary driver of household displacement outcomes. However, various additional factors influence household return. Some factors are globally important (i.e., for all households in the model), such as property damage, unsanitary conditions after the disaster, tenure status, and the income per household member. Other factors had lower global importance but significant local importance (i.e., for some households in the model), such as household sizes of 8+, educational attainment less than high school, and physical immobility. The importance of each feature also varies depending on the displacement class; for example, tenure status was more important for the recovery phase displacement and not returned classes than for the emergency phase displacement class.

These proposed classification tree models were applied to a hurricane scenario analysis for Atlantic City, NJ: one with socioeconomic factors (TreeP&S) and one with physical factors only (TreeP). Although both models yielded consistent estimates for the percentage of households displaced in the emergency phase, the physical factors‐only model (TreeP) was unable to distinguish between households displaced in the recovery phase versus those that did not return. The implication is that some socioeconomic factors require consideration to differentiate between long‐term displacement and permanent relocation.

As for policy implications, the proposed predictive models can, for instance, directly support a quantitative evaluation of the potential benefit of physical retrofit strategies via the property damage predictor. While some other key soft policy factors are missing from the HPS dataset (e.g., insurance status and post‐disaster assistance received), the model explanations highlight population subgroups that have experienced disproportionate long‐term displacement and potential permanent relocation risk in recent disasters (e.g., non‐homeowners, low‐income households, those with physical immobility, large households, and those with low educational attainment levels) and would thus benefit most from improved targeting or support. Ideally, future iterations of the HPS will refine questions related to long‐term displacement and permanent relocation to better inform such policy decisions and evaluations.

## Figures and Tables

**FIGURE A1:**
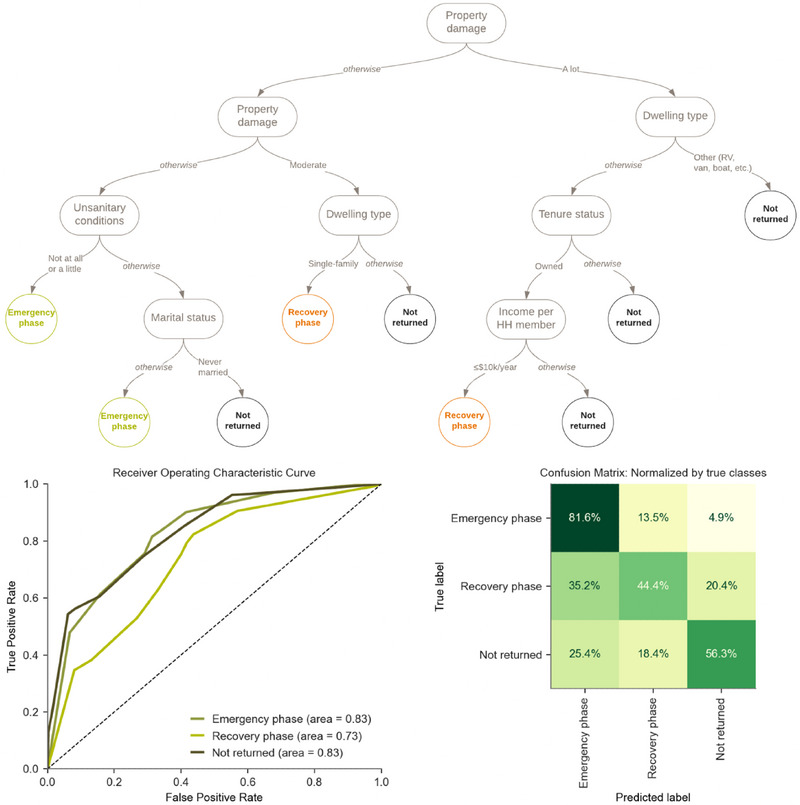
The receiver operating characteristic (ROC) curves and the confusion matrix for top scoring but more complex TreeP&S model variant.

## Data Availability

Appendix A defines the data sources used to fit the predictive models. All input data are publicly available from the listed sources. The code used to fit and evaluate the predictive models is available in a public GitHub repository: https://github.com/nicolepaul/hps‐displacement. A simple dashboard to visualize and interact with the HPS data is available at: https://hps.nicolepaul.io/.

## APPENDIX A DATA DICTIONARY

This appendix provides the data dictionary for all explanatory variables considered within the proposed models for household displacement durations. All household‐level data came directly from the Public Use Files (PUFs) associated with the United States Household Pulse Survey (HPS). This data source primarily covers physical damage to the built environment (see Table A1) and household demographics (see Table A2). However, information related to other factors that can influence household return, such as psychological and social phenomena or pre‐ and post‐disaster policies, was not available in the HPS. To cover these topics, area attributes (i.e., at the Metropolitan Statistical Area or the state level) were included based on additional data sources, as summarized in Table A3.

**TABLE A1:** The explanatory variables considered for the household displacement duration model: Physical damage to the built environment.

Explanatory variable	Data type	Values	Source
Property damage	Ordinal	No damage Some damage Moderate amount of damage A lot of damage	Household Pulse Survey
Water shortage after disaster	Ordinal	Not at all A little Some A lot	Household Pulse Survey
Electricity loss after disaster	Ordinal	Not at all A little Some A lot	Household Pulse Survey
Unsanitary conditions after disaster	Ordinal	Not at all A little Some A lot	Household Pulse Survey
Food shortage after disaster	Ordinal	Not at all A little Some A lot	Household Pulse Survey
Hazard type	Nominal	Hurricane Flood Fire Tornado Other Multiple	Household Pulse Survey
Dwelling type	Nominal	Single‐family Multi‐family Mobile home Other (boat, RV, van, etc.)	Household Pulse Survey

**TABLE A2:** The explanatory variables considered for the household displacement duration model: Household demographics.

Explanatory variable	Data type	Values	Source
Tenure status	Nominal	Owned Rent Occupied without payment	Household Pulse Survey
Household size	Ordinal	1 2 3–4 5–7 8+	Household Pulse Survey
School enrollment	Nominal	None Public school Private school Public and private school	Household Pulse Survey
Income per household member	Ordinal	Less than $10,000 $10,000–$19,999 $20,000–$29,999 $30,000–$49,999 $50,000–$99,999 $100,000–$149,999 $150,000 and above	Household Pulse Survey
Race	Nominal	White Black Asian Other/Mixed	Household Pulse Survey
Marital status	Nominal	Now married Widowed Divorced Separated Never married	Household Pulse Survey
Hispanic origin	Nominal	Yes No	Household Pulse Survey
Employment status	Nominal	Yes No	Household Pulse Survey
Age	Ordinal	24 or less 25–34 35–44 45–54 55–64 65–74 75+	Household Pulse Survey
Educational attainment	Ordinal	Less than high school Some high school High school or equivalent Some college Associate's degree Bachelor's degree Graduate degree	Household Pulse Survey
Limited mobility	Ordinal	No—no difficulty Yes—some difficulty Yes—a lot of difficulty Cannot do at all	Household Pulse Survey

**TABLE A3:** The explanatory variables considered for the household displacement duration model: Area attributes.

Explanatory variable	Data type	Values	Source
Unemployment rate (2022)	Continuous	Min: 2.1% Median: 3.4% Max: 5.4%	Bureau of Labor Statistics
Disaster declarations (2021–2023)	Continuous	Min: 0 Median: 109 Max: 277	OpenFEMA
Homeownership rate (2022)	Continuous	Min: 48.3% Median: 67.3% Max: 78.6%	American Housing Survey
Homeowner vacancy rate (2022)	Continuous	Min: 0.3% Median: 0.8% Max: 2.1%	American Housing Survey
Rental vacancy rate (2022)	Continuous	Min: 2.5% Median: 6.9% Max: 12.2%	American Housing Survey
Population change (2010–2020)	Continuous	Min: −3.4% Median: 7.5% Max: 20.0%	US Census Bureau

For reference, the relevant links for each data source are provided below:

- **Household Pulse Survey**: https://www.census.gov/programs‐surveys/household‐pulse‐survey.html
- **Bureau of Labor Statistics** (Unemployment rates): https://www.bls.gov/lau/
- **OpenFEMA** (Disaster declarations): https://www.fema.gov/openfema‐data‐page/disaster‐declarations‐summaries‐v1
- **American Housing Survey** (Rental vacancy rates, homeowner vacancy rates, homeownership rates): https://www.census.gov/programs‐surveys/ahs.html
- **US Census Bureau** (Population estimates): https://www.census.gov/programs‐surveys/popest/data/data‐sets.html

## APPENDIX B MODEL PERFORMANCE COMPARISONS

### B.1

#### Hyperparameter parameters and cross‐validation results

The considered hyperparameters, hyperparameter descriptions, and cross‐validation results are summarized in Tables A4 and A5 for the TreeP&S and the ForestP&S model, respectively.

**TABLE A4:** Hyperparameter values considered for the classification tree model (TreeP&S).

Hyperparameter	Elaboration	Considered values
Maximum depth	The maximum depth of the classification tree, where a smaller depth indicates a simpler model. Practical heuristics for selecting the maximum depth include the logarithm of the number of explanatory variables considered (∼3) or the square root of the number of explanatory variables considered (∼5).	3, 4^a^, and 5
Cost complexity parameter, α	Cost complexity pruning combats overfitting by preventing the tree from splitting further branches unless node impurity is improved beyond a given threshold, as specified by the parameter α.	0.1%, 0.2%, 0.3%, and 0.4%^a^
Maximum leaf nodes	The maximum number of leaf nodes (i.e., terminal nodes). Low values restrict tree growth, leading to a simpler model. However, small values can lead to underfitting.	6, 8, and 10^a^
Minimum samples per leaf	If a split will result in a child node with fewer than this number of samples, the split is avoided. Lower values can lead to overfitting with high variance, whereas higher values can lead to a high bias. Since the data have been expanded by the household weights (50 times or more), relatively high values are considered here.	1, 500, 2000, 5000^a^, and 20,000

^a^ The selected values in the final model.

**TABLE A5:** Hyperparameter values considered for the random forest model (ForestP&S).

Hyperparameter	Elaboration	Considered values
Maximum depth	See description in Table A4.	3, 4, and 5
Cost complexity parameter, α	See description in Table A4.	0.1%, 0.2%, 0.3%, and 0.4%
Maximum leaf nodes	See description in Table A4.	6, 8, and 10
Minimum samples per leaf	See description in Table A4.	1, 500, 2000, 5000, and 20,000
Number of estimators	Each random forest model is a collection of estimators (in this case, individual classification tree models). The resulting classification is determined by majority rule across all estimators.	300, 500, 700, and 900^a^

^a^The selected values in the final model.

#### Performance of alternative models

##### Proposed models

A summary of the performance of the proposed models on the testing set is in Table A6.

**TABLE A6:** Performance of the proposed models on the testing set.

Evaluation metric		TreeP&S	ForestP&S	TreeP
AUC	Emergency phase	0.83	0.86	0.83
	Recovery phase	0.73	0.77	0.75
	Not returned	0.83	0.86	0.79
	Macro average	0.80	0.83	0.79
F1 Score	Emergency phase	0.84	0.84	0.78
	Recovery phase	0.45	0.46	0.33
	Not returned	0.51	0.48	0.43
	Macro average	0.60	0.59	0.51

Abbreviation: AUC, area under curve.

##### Alternate machine learning algorithms

A variety of common supervised machine learning algorithms were initially investigated in this study, with results prior to hyperparameter tuning summarized in Table A7. Except for K Nearest Neighbors and Naïve Bayes, the preliminary results are largely comparable.

**TABLE A7:** Performance comparison across alternate machine learning algorithms during initial model fitting.

Machine learning algorithm	Method (sklearn)	Hyperparameters	Calculation time	AUC		
				Emergency phase	Recovery phase	Not returned
Logistic regression	LogisticRegression	penalty = “L2”	3 min 35 s	0.86	0.75	0.84
Classification tree	DecisionTreeClassifier	max_depth = 5	5 s	0.85	0.75	0.79
Random forest	RandomForestClassifier	max_depth = 5 n_estimators = 350	1 min 33 s	0.86	0.77	0.86
Support vector machine	SVC	kernel = “linear”	Did not complete in 130 h	–	–	–
Support vector machine	LinearSVC	–	41 s	0.85	0.74	0.84
K nearest neighbors	KNeighborsClassifier	n_neighbors = 146	1 min 37 s	0.74	0.62	0.65
Naïve Bayes	GaussianNB	–	23 s	0.80	0.70	0.81

Abbreviation: AUC, area under curve.

##### Alternate TreeP&S hyperparameters

Table A8 summarizes the cross‐validation results for the top 10 best performing hyperparameter configurations for the TreeP&S model. The average score (i.e., the macro‐average of the AUC and F1 across all classes), consistency (i.e., standard deviation), and complexity (i.e., number of predictors required) were all considered before identifying the proposed model.

**TABLE A8:** Performance comparison across alternate TreeP&S models during cross‐validation; the selected model highlighted in bold.

Rank	Hyperparameters	Score (testing)	Score (training)	Consistency	Complexity
1	ccp_alpha = 0.003 max_depth = 4 min_samples = 20,000 max_leaf_nodes = 10	0.677	0.679	0.011	Property damage, dwelling type, tenure status, income per household member, unsanitary conditions, and marital status.
2	ccp_alpha = 0.003 max_depth = 4 min_samples = 5000 max_leaf_nodes = 10	0.677	0.680	0.012	Property damage, dwelling type, tenure status, income per household member, unsanitary conditions, and marital status.
3	ccp_alpha = 0.003 max_depth = 5 min_samples = 5000 max_leaf_nodes = 10	0.676	0.680	0.011	Property damage, dwelling type, tenure status, unsanitary conditions, electricity loss, marital status, and hazard type.
4	ccp_alpha = 0.003 max_depth = 5 min_samples = 20,000 max_leaf_nodes = 10	0.676	0.679	0.010	Property damage, dwelling type, tenure status, unsanitary conditions, electricity loss, marital status, and hazard type.
5	ccp_alpha = 0.004 max_depth = 5 min_samples = 20,000 max_leaf_nodes = 10	0.673	0.676	0.010	Property damage, dwelling type, tenure status, unsanitary conditions, electricity loss, and hazard type.
6	ccp_alpha = 0.004 max_depth = 5 min_samples = 5000 max_leaf_nodes = 10	0.673	0.677	0.012	Property damage, dwelling type, tenure status, unsanitary conditions, electricity loss, and hazard type.
7	ccp_alpha = 0.004 max_depth = 4 min_samples = 5000 max_leaf_nodes = 10	0.669	0.673	0.011	Property damage, dwelling type, and tenure status.
8	ccp_alpha = 0.004 max_depth = 4 min_samples = 20,000 max_leaf_nodes = 10	0.668	0.670	0.010	Property damage, dwelling type, and tenure status.
9	ccp_alpha = 0.004 max_depth = 5 min_samples = 20,000 max_leaf_nodes = 8	0.667	0.669	0.008	Property damage, dwelling type, and tenure status.
10	ccp_alpha = 0.004 max_depth = 4 min_samples = 20,000 max_leaf_nodes = 8	0.666	0.667	0.010	Property damage, dwelling type, and tenure status.

Some more complex models outperformed the proposed TreeP&S model, but only slightly. The top‐performing model is shown in Figure A1 for reference. This model variant additionally would require the following predictors: unsanitary conditions after the disaster, the income level per household member, and the marital status of the household.

##### Alternate ForestP&S hyperparameters

Table A9 summarizes the cross‐validation results for the top 10 best performing hyperparameter configurations for the TreeP&S model. The average score (i.e., the macro‐average of the AUC and F1 across all classes), consistency (i.e., standard deviation), and concerns about overfitting (i.e., the hyperparameters n_estimators and min_samples_leaf) were all considered before identifying the proposed model.

**TABLE A9:** Performance comparison across alternate ForestP&S models during cross‐validation; the selected model highlighted in bold.

Rank	Hyperparameters	Score (testing)	Score (training)	Consistency
1	ccp_alpha = 0.001 max_depth = 5 min_samples_leaf = 1 n_estimators = 700	0.760	0.766	0.012
2	ccp_alpha = 0.001 max_depth = 5 min_samples_leaf = 500 n_estimators = 700	0.759	0.765	0.012
3	ccp_alpha = 0.001 max_depth = 5 min_samples_leaf = 500 n_estimators = 300	0.759	0.765	0.011
4	ccp_alpha = 0.001 max_depth = 5 min_samples_leaf = 1 n_estimators = 300	0.759	0.765	0.011
5	ccp_alpha = 0.001 max_depth = 5 min_samples_leaf = 1 n_estimators = 500	0.759	0.765	0.012
6	ccp_alpha = 0.001 max_depth = 5 min_samples_leaf = 500 n_estimators = 500	0.759	0.765	0.011
7	ccp_alpha = 0.001 max_depth = 5 min_samples_leaf = 2000 n_estimators = 500	0.754	0.760	0.011
8	ccp_alpha = 0.001 max_depth = 5 min_samples_leaf = 2000 n_estimators = 700	0.753	0.759	0.011
9	ccp_alpha = 0.001 max_depth = 5 min_samples_leaf = 2000 n_estimators = 300	0.752	0.759	0.011
10	ccp_alpha = 0.001 max_depth = 5 min_samples_leaf = 5000 n_estimators = 500	0.743	0.749	0.011
